# Impact of a care process model on outcomes in emergency department patients with atrial fibrillation

**DOI:** 10.1016/j.hroo.2025.07.011

**Published:** 2025-07-24

**Authors:** Rahul Ahuja, Michael Von Bargen, Howard Klemmer, Daniel Cheng, Mahdi Veillet-Chowdhury, Todd Seto, Bert Matsuo, Kailie Wong, Sara Hamele, David Singh

**Affiliations:** Center for Heart Rhythm Disorders, The Queens Heart Institute, Queen’s Medical Center, Honolulu, Hawaii

**Keywords:** Atrial fibrillation, Emergency department, Care process model, Oral anticoagulation, Best practice alert, CHA_2_DS_2_-VASc, Quality improvement

## Abstract

**Background:**

Atrial fibrillation (AF) in the emergency department (ED) is a growing public health burden, marked by significant variability in management, particularly regarding oral anticoagulation (OAC). Care process models (CPMs), supported by real-time decision tools, may improve standardization and outcomes.

**Objective:**

This study aimed to evaluate the impact of a CPM on clinical outcomes, treatment patterns, and documentation for patients with AF presenting to EDs within a large integrated health care system.

**Methods:**

We implemented a CPM in the Queen’s Health Systems (Hawaii), targeting ED patients with AF. Interventions included a structured treatment algorithm, an AF response team, best practice alerts (BPAs) for OAC and CHA_2_DS_2_-VASc documentation, and near-real-time data monitoring. Outcomes were assessed across 3 phases: pre-CPM, post-CPM/pre-BPA, and post-BPA. Primary outcomes included OAC compliance, documentation of OAC contraindications, cardioversion rates, length of stay (LOS), and admission rates.

**Results:**

Among 3236 patients with AF (2020–2025), OAC compliance improved from 60.1% to 72.1% after BPA (P < .00001) and to 83% when excluding those with OAC contraindications. CHA_2_DS_2_-VASc documentation increased from 5% to 40% (P < .00001). Cardioversion rates increased from 11.9% to 16.8% (P < .0001). Hospital admissions declined from 44% to 38% (P = .004). ED LOS for discharged patients increased slightly (3.6–4.0 hours, P = .0005); inpatient LOS remained stable.

**Conclusion:**

System-wide CPM implementation improved OAC use, documentation, and cardioversion rates, while reducing admissions. Despite a modest increase in ED LOS, the model supported more consistent, guideline-based AF care, reinforcing the value of multidisciplinary, algorithm-driven strategies in emergency settings.

**Key Findings**

▪ A well-designed care process model (CPM) can meaningfully influence physician behavior in the emergency department, leading to more standardized care for patients with atrial fibrillation (AF).
▪ The CPM implementation was associated with a significant increase in oral anticoagulation prescribing rates, improving to 83% among eligible patients after excluding those with documented contraindications.
▪ Cardioversion rates modestly increased after implementation, reflecting improved physician confidence and access to structured follow-up care.
▪ CHA_2_DS_2_-VASc score documentation improved significantly through the use of a best practice alert, enhancing risk stratification at the point of care.
▪ Hospital admission rates for patients with AF decreased after CPM, suggesting that improved frontline decision making and reliable follow-up pathways can safely reduce inpatient utilization.

## Introduction

Atrial fibrillation (AF) remains the most common arrhythmia in adults and is associated with considerable morbidity and mortality. The incidence of AF continues to increase worldwide and is projected to affect 12.1 million people in the United States (US) by 2050 and 17.9 million people in Europe by 2060.^1^ AF imposes a substantial financial burden on the health care system, with total annual costs in the US estimated to range between $6 billion and $26 billion.^2^ A growing share of this cost is borne by emergency departments (EDs), where AF-related visits increased by more than 30% between 2007 and 2014, with associated annual charges for admitted patients rising from $7.39 billion to $10.1 billion.^3^ In addition, recent analyses have shown that the cost of care for an AF-related ED visit resulting in admission can exceed $9000 per encounter.^4^ A large proportion of patients with AF presenting to the ED in the US are admitted to the hospital, contributing further to the financial burden on patients and health systems.^3^

Along with health care costs, multiple large-scale studies have shown gaps in the prescription of oral anticoagulants (OACs) to patients with appropriately elevated CHA_2_DS_2_-VASc scores in the ED.^5^, ^6^, ^7^, ^8^ These gaps may be attributed to a lack of documented reasons for deferral, insufficient standardization in care delivery models, patient-related factors, and discomfort among physicians in making an OAC decision for a patient they will not be subsequently treating^9^^,^^10^ Decisions related to management, including rate/rhythm control, choice of anticoagulation, and inpatient admission criteria, are influenced heavily by an ED provider’s comfort level. Suboptimal anticoagulation is a well-established contributor to preventable thromboembolic events, which can drive higher admission rates, resource use, and downstream health care costs. The ED setting represents a critical opportunity to address this gap, particularly for patients who may not otherwise have timely outpatient follow-up.

Care process models (CPMs) are structured, evidence-based frameworks designed to standardize workflow, reduce provider burden, and address care gaps across various clinical settings.^9^, ^10^, ^11^, ^12^ Frequently, CPMs incorporate real-time tools such as computerized decision support or best practice alerts (BPAs) to assist providers in making guideline-concordant treatment decisions. These tools have been shown to improve prescribing practices for OACs in both outpatient and ED settings.^8^^,^^10^^,^^13^

Beyond their impact on specific prescribing behaviors, CPMs contribute to broader improvements in health care delivery—promoting adherence to clinical guidelines, facilitating timely and appropriate referrals, enhancing interdepartmental communication, and supporting fiscally responsible care models. Moreover, close collaboration between electrophysiology (EP) and ED providers within a CPM framework may further help reduce length of stay (LOS), optimize acute management, decrease admission rates, and lower the overall cost of care.

In this study, we present the impact of a custom-tailored AF CPM designed to standardize the treatment of AF across the largest health care system in Hawaii. The Queen’s Health Systems (QHS) encompasses 4 major hospitals: The Queen’s Medical Center, a tertiary care facility with 575 beds; The Queen’s Medical Center West O’ahu, with approximately 104 beds; Molokai General Hospital, with approximately 15 beds; and Queen’s North Hawaii, with approximately 35 beds. Each hospital is equipped with its own emergency room.

## Methods

**Figure 1:**
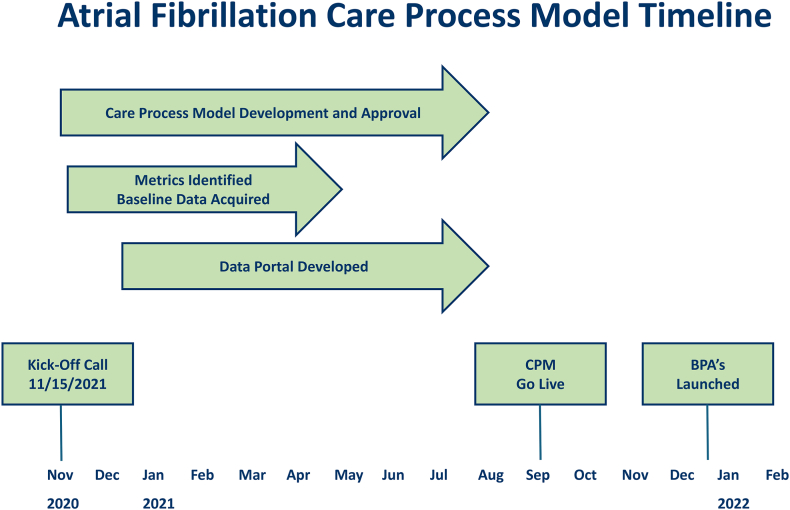
Timeline for development and implementation of atrial fibrillation CPM. BPA = best practice alert; CPM = care process model.

In November 2020, we began the development of a CPM focused on patients with AF admitted to the emergency rooms in the QHS. A timeline denoting the key milestones in the development and implementation of the CPM is presented in Figure 1. Key stakeholders were invited to meet weekly and included electrophysiologists, EP advanced practice registered nurses (APRNs), ED physicians from participating Queen’s campuses, ED administrative personnel, electronic health records (EHRs) staff (Epic), and members of Queen’s data analytics teams. This working group agreed upon a plan of action that included developing key quality metrics around which the CPM would be designed. All metrics were assessed with respect to patients admitted to a QHS ED presenting with a primary diagnosis of AF or atrial flutter (AFL). These metrics included the percentage of patients who underwent direct current cardioversion in the ED, percentage of patients who were discharged on appropriate anticoagulation, percentage of patient who were admitted from ED, percentage of patients for whom a CHA_2_DS_2_-VASc score was documented in the medical record, ED and inpatient LOS, and response time from the AF response ream (described below).

To track our key metrics, a secure, Web-based data analytics portal was developed. This portal provided near-real-time access to CPM metrics. The portal was accessible to authorized health care providers and administrative staff through the QHS intranet, ensuring timely visibility into patient-level and aggregate performance data. By pulling directly from the EHR system, the portal automatically refreshed key indicators, enabling data-driven decision making and rapid identification of improvement opportunities. This approach streamlined monitoring efforts, supported continuous quality improvement, and fostered transparency among stakeholders.

**Figure 2:**
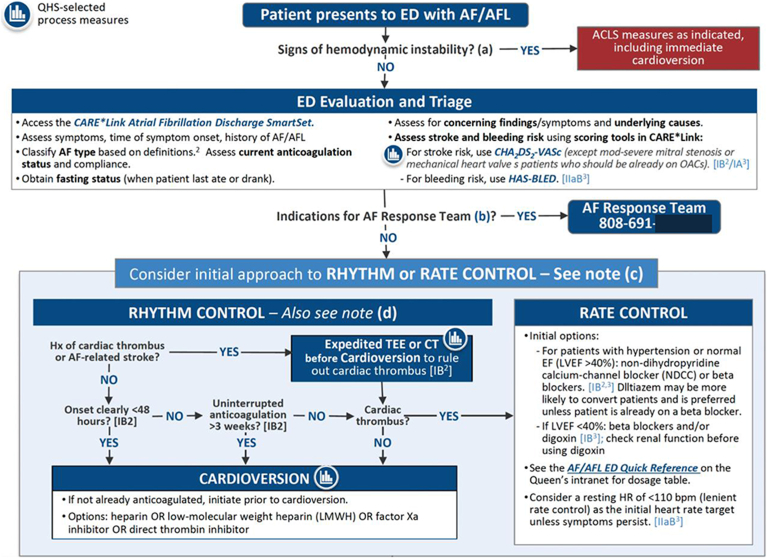
Algorithm for ED care process model. ACLS = advanced cardiac life support; AF = atrial fibrillation; AFL = atrial flutter; ED = emergency department; LVEF = left ventricular ejection fraction.

An algorithm was developed for patients with a primary diagnosis of AF presenting to EDs in the QHS (Figure 2). The algorithm began with an initial evaluation conducted by an ED provider to identify AF/AFL via electrocardiogram. Initial assessment also included evaluation of hemodynamic stability, evaluation of stroke and bleeding risks, and identification of factors that favored either rate or rhythm control.

Central to the algorithm was the establishment of an AF response team consisting of 4 trained EP APRNs (one of whom is designated to respond to AF response team activations on any given day) and 1 supervising EP physician. The team was available Monday to Friday from 7:30 AM to 4:00 PM. Afterhours, weekends, and holidays were covered by the cardiology fellows on call who were also trained in the algorithm. Criteria for the activation of the AF response team included new-onset AF or AFL, the need for expedited cardioversion and/or transesophageal echocardiogram, consideration of pharmacologic rhythm control, and uncertainty regarding anticoagulation. Once the team was activated, response times were tracked from initial contact to bedside assessment by the APRN. The EP APRN collaborated closely with patients, ED providers, and the EP attending physicians to determine the optimal course of action. One key element of the algorithm was that any patient discharged with a primary diagnosis of AF could be seen in a dedicated AF clinic within 7 days after discharge.

**Figure 3:**
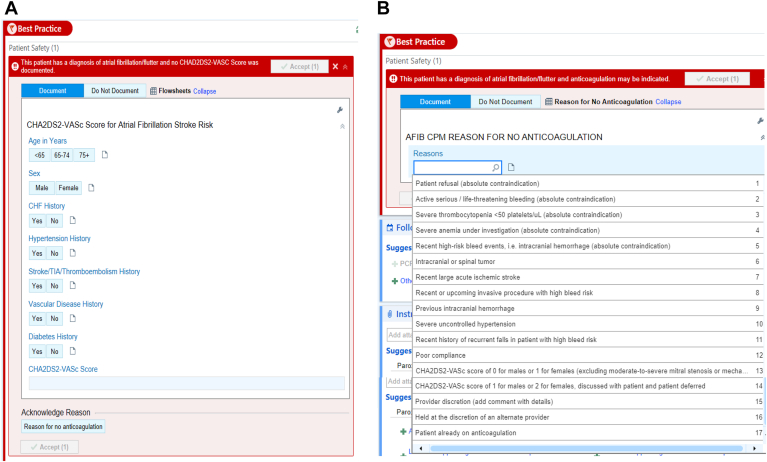
**A:** Best practice alert: a passive alert appeared when a patient with atrial fibrillation and no documented OAC and/or CHA_2_DS_2_-VASc score was identified. These data were directly exported to the data analytics portal. **B:** An additional best practice alert was triggered when a patient with atrial fibrillation was not on OAC. It allowed the provider to document contraindications to OAC via a dropdown menu. These data were directly exported to the data analytics portal. AFIB = atrial fibrillation; CHF = congestive heart failure; CPM = care process model; OAC = oral anticoagulant; TIA = transient ischemic attack.

To support the appropriate initiation of anticoagulation therapy, we implemented 2 passive BPAs. The first prompted providers to document a CHA_2_DS_2_-VASc score for patients in whom one had not yet been recorded (Figure 3A). The second alerted providers when a patient with AF/AFL was not currently prescribed anticoagulation. In instances where OAC was contraindicated, a dropdown menu within the BPA provided a user-friendly approach for documentation that was connected to the data portal (Figure 3B). The latter was important to determine an accurate assessment of patients who were deemed to be “compliant” with respect to the anticoagulation CPM metric. In this study, anticoagulation compliance was defined as the appropriate prescription of anticoagulants for AF or AFL in patients without contraindications, and a CHA_2_DS_2_-VASc score of ≥2. For this study, the term “contraindication” encompasses both clinical considerations (eg, fall risk, bleeding risk) and patient-directed decisions (eg, refusal), recognizing that although patient refusal may not constitute a strict medical contraindication, it often results from shared decision making and meaningfully affects prescribing behavior in real-world practice.

To assess the effect of the CPM on OAC prescribing rates, we analyzed compliance rates across 3 phases: phase 1 (pre-CPM), phase 2 (post-CPM/pre-BPA), and phase 3 (post-BPA). Both unadjusted OAC compliance rates (including all eligible patients) and adjusted compliance rates (excluding patients with OAC contraindications) were examined. The impact of the CPM on other metrics was assessed by comparing baseline data (pre-CPM) with a singular post-CPM phase. In addition to the metrics outlined earlier, we also chose to examine the impact of the CPM on 30-day readmission rates and hospital LOS for patients who were admitted.

**Figure 4:**
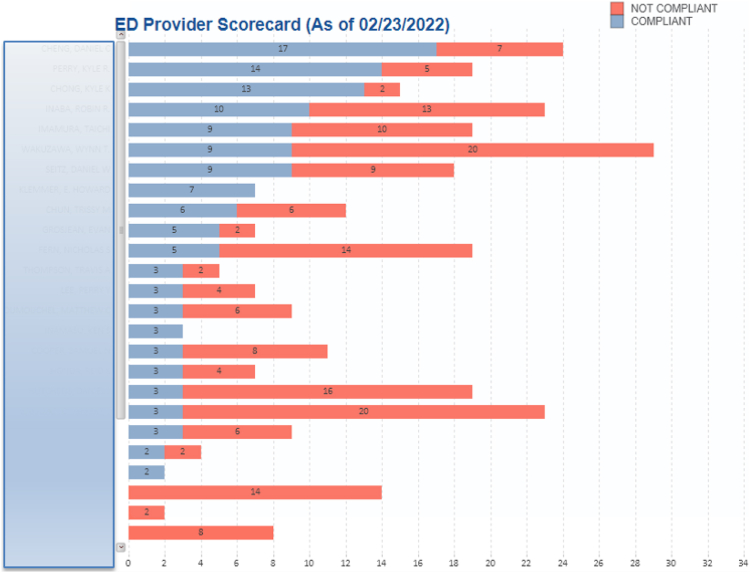
A physician report card indicating that physician-level compliance with the care process model was periodically presented at ED meetings. Physician names were not deidentified but have been obscured in the figure for privacy. ED = emergency department

Several measures were used to promote the adoption and sustained usage of the CPM. In the preimplementation phase, several meetings were held among stakeholders including ED physicians and staff, members of the cardiology department, key pharmacy personnel, and others to make them aware of the project and the proposed implementation date. After the CPM “go-live” date, periodic updates were provided to these stakeholders regarding compliance with the various CPM metrics. A provider scorecard was also shared monthly, which outlined provider-level compliance with the CPM (Figure 4). Compliance was defined as any patient discharged from the ED with a documented CHA_2_DS_2_-VASc score, appropriate anticoagulation prescribed when indicated, and documented contraindications to OAC when applicable. In addition, automated e-mails were sent to providers when noncompliant treatment was posted to the data portal. These e-mails served as a gentle reminder to encourage adherence to the CPM for future encounters.

Institutional review board approval was not required for this study, given that it used deidentified data collected for ongoing surveillance of the CPM as part of a system-wide quality improvement initiative.

### Statistical analysis

Statistical analyses were performed in JMP (SAS Institute, Cary, NC). Data from January 1, 2020, to January 31, 2025, were included. Categorical variables (eg, OAC compliance, admission, and readmission rates) are reported as counts and percentages, whereas continuous variables (eg, ED LOS, CHA_2_DS_2_-VASc scores) are summarized as means ± standard deviation when normally distributed or medians (interquartile range) when skewed; normality was assessed with Shapiro–Wilk tests. Between-phase comparisons used χ^2^ tests for categorical outcomes and independent-samples t tests for normally distributed continuous measures, substituting Wilcoxon rank-sum tests when normality assumptions were violated. Cardioversion rate changes were evaluated with 2-proportion z-tests. Records with missing key variables (<5% overall) were excluded from the relevant analyses; no imputation was performed. All tests were 2 sided, with P < .05 indicating statistical significance.

## Results

A total of 3236 patients with a primary diagnosis of AF were seen and evaluated in QHS emergency rooms between January 2020 and January 2025 (Table 1). The mean age of patients for the pre- and post-CPM phases was approximately 66 and 67 years, respectively. When the AF team was contacted, the median response time from the time of the call to first contact with the patient in the ED was 4 minutes.

**Table 1:** Baseline characteristics of patients in the baseline and post-care process model groups

Characteristic	Baseline group (n = 902)	After CPM (n = 2334)
Age (mean ± SD)	66.4 ± 14.2	67.1 ± 14.1
Sex		
Female	350 (38.8)	993 (42.5)
Male	552 (61.2)	1341 (57.5)
Race/ethnicity		
White	350 (37.3)	937 (40.1)
Asian	322 (35.7)	812 (34.8)
Native Hawaiian or other Pacific Islander	207 (22.9)	526 (22.5)
Black	21 (2.3)	31 (1.3)
Other	16 (1.8)	28 (1.2)
CHA_2_DS_2_-VASc		
Mean CHA_2_DS_2_-VASc, female	3.4	3.2
Mean CHA_2_DS_2_-VASc, male	1.3	1.8
Patients with documented CHA_2_DS_2_-VASc, n (%)	47/902 (5)	942/2334 (40)
CHA_2_DS_2_-VASc of ≤1	2.20%	10.70%

CPM = care process model; SD = standard deviation.

### Impact of documenting OAC contraindications on OAC compliance

**Figure 5:**
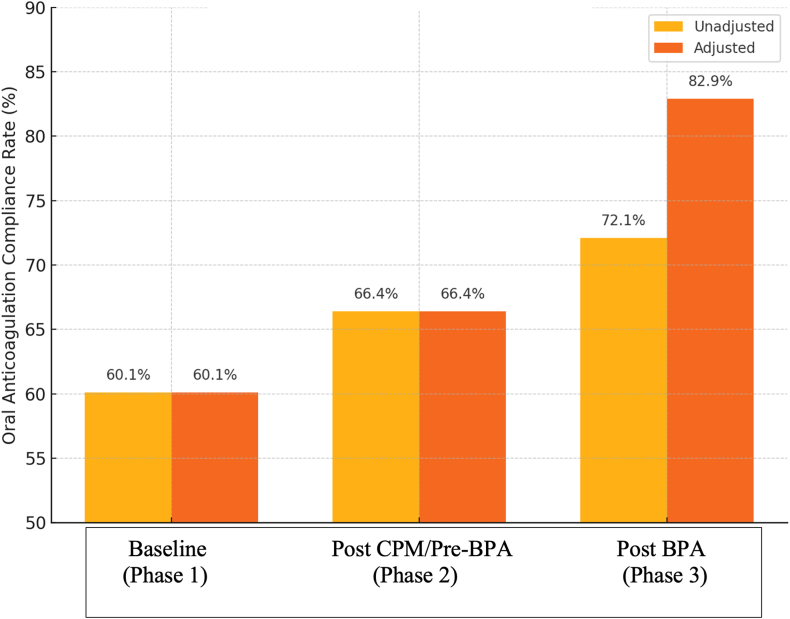
Oral anticoagulation (OAC) compliance rates across study phases, unadjusted vs adjusted for contraindications. This figure compares OAC compliance rates (unadjusted) and adjusted OAC compliance rates (patients with OAC contraindications excluded) across 3 study phases. Compliance rates improved over time, with the greatest increase observed in the post-BPA phase after accounting for contraindications. Percentages reflect observed rates within each phase (unadjusted, phase 1 vs phase 2 [P = .061], baseline vs phase 3 [P < .00001], phase 2 vs phase 3 [P = .045]; adjusted, phase 1 vs phase 2 [P = .061], phase 1 vs phase 3 [P < .00001], phase 2 vs phase 3 [P < .00001]). BPA = best practice alert; CPM = care process model.

There were no documented OAC contraindications in either phase 1 (pre-CPM) or phase 2 (post-CPM/pre-BPA). During phase 3 (post-BPA), 95 patients (11%) were found to have documented contraindications to OAC and were therefore excluded from analysis. The most frequent documented contraindications were provider discretion (41%), patient refusal (15%), and a history of falls (13%). When patients with documented contraindications to anticoagulation were excluded from the analysis, the OAC compliance rate improved to 83% (P < .00001) (Figure 5).

### Impact of the CPM on cardioversion rates and documentation of CHA_2_DS_2_-VASc score

After the CPM implementation, there was a significant improvement in the percentage of patients undergoing cardioversion in the ED (Table 2). The cardioversion rate for patients presenting with a primary diagnosis of AF increased from 11.9% during the pre-CPM phase to 16.8% in the post-CPM phase (P < .0001). CHA_2_DS_2_-VASc documentation in the centralized region of the EHR also improved in the post-CPM phase. During the pre-CPM phase, 5% of patients were found to have a documented CHA_2_DS_2_-VASc score (n = 47 of 902). During the post-CPM phase, this percentage increased to 40% (n = 942 of 2334, P < .00001) (Table 2).

**Table 2:** Comparison of key outcomes: baseline vs after CPM

Care process model metric	Baseline	After CPM	P value
Cardioversion rate, number of patients (%)	108/902 (11.9%)	392/2334 (16.8%)	<.0001
CHA_2_DS_2_-VASc documentation	47/902 (5)	942/2334 (40)	<.00001
Admission rate, number of patients (%)	393/902 (44)	824/2187 (38)	.002
30-d readmission rate, number of patients (%)	5/456 (1.1)	8/824 (0.97)	1.00
ED LOS: discharged patients (h), mean	3.59	3.95	.005
ED LOS: admitted patients (h), median	6.4	8.3	.37
Hospital LOS (d), median	2.15	2.13	.98

CPM = care process model; ED = emergency department; LOS = length of stay.

### Impact of CPM on admission and readmission rates

Admission rates for patients presenting to QHS EDs were reduced after the CPM implementation. The admission rate during the pre-CPM phase was 44% (corresponding to a discharge rate of 56%). After the CPM implementation, the admission rate for this cohort was reduced to 38% (corresponding to a discharge rate of 62%) (P = .004) (Table 2). There was no significant difference in 30-day readmission rates between pre- and post-CPM phases (1.01% vs 1.02%, P = 1.0).

### Impact on LOS

The CPM implementation had a significant impact on ED LOS for patients who were discharged from the ED. The ED LOS for patients increased from 3.6 to 4.0 hours from the pre-CPM phase to the post-CPM phase (P = .0005) (Table 2).

There was a nonsignificant trend toward increased LOS for patients presenting with a primary diagnosis of AF who were subsequently admitted to the hospital. The ED LOS was 6.4 hours during the pre-CPM phase and 8.3 hours in the post-CPM phase (P = .37). There was no significant difference in hospital LOS among patients who were evaluated in the ED and subsequently admitted to the hospital. The hospital LOS was 2.15 days during the pre-CPM phase and 2.13 days during the post-CPM phase (P = .98).

## Discussion

The findings of this study support the concept that a CPM implementation in a large health system can have a meaningful impact on the treatment of AF. Over the decades, CPMs have gained widespread implementation, particularly with the advent of EHRs. Notable examples include the “Surviving Sepsis Campaign,” which introduced sepsis care bundles in infectious disease management, and the enhanced recovery after surgery protocols in orthopedics, which have significantly improved outcomes for patients undergoing total joint replacement surgeries.^4^^,^^14^ Similarly, in cardiology, CPMs have demonstrated their utility through initiatives such as heart failure management programs, which enhance adherence to guideline-directed medical therapy, and acute myocardial infarction care pathways that streamline treatment to improve survival rates.^15^^,^^16^

There have been several professional groups that have proposed algorithm-based approaches for AF including the Heart Rhythm Society and the American College of Emergency Physicians. In addition, the most recent US AF guidelines give the utilization of clinical care pathways for the management of AF a 2a recommendation (level of evidence B-R).^17^ Our study aimed to build upon the existing evidence supporting the efficacy of CPMs, specifically in the management of AF/AFL within the ED setting. Even though AF was a common presentation in EDs throughout our system, there was no standardized approach to its management.

To address this gap, we developed and implemented a structured care pathway that leveraged a team-based approach to streamline management and improve outcomes. Key components of this pathway include prompt evaluation by the AF response team, adoption of an appropriate treatment strategy, appropriate administration of OAC, systematic documentation of contraindications for OAC, and expedited cardioversions when appropriate.

Although AF management encompasses a wide range of strategies, including risk factor modification, long-term rhythm control, and rate management, we elected to focus primarily on OAC within our CPM owing to the specific context and constraints of the ED setting. These metrics were selected a priori during CPM development, based on stakeholder consensus regarding which interventions were both feasible and impactful in the acute care environment.

In the preimplementation phase of the CPM, the baseline OAC utilization among eligible patients with AF was 60.1%. This finding is consistent with other studies that have examined OAC prescription rates for patients with AF in both inpatient and outpatient settings. Several observational studies have documented OAC utilization rates in these settings ranging from 45% to 64%.^5^^,^^18^^,^^19^

Studies focusing specifically on ED settings revealed baseline OAC rates for patients with AF ranging from 37% to 66%.^6^, ^7^, ^8^ These ED-based studies also implemented quality-driven interventions aimed at enhancing OAC discharge rates for patients with AF, with 2 of the 3 demonstrating significant improvements.^7^^,^^8^

Our findings demonstrate that the implementation of a well-designed CPM can be associated with a significant increase in appropriate OAC prescription rates. Our data suggest that the increase in OAC compliance was driven by both increased prescriber awareness and the standardized documentation of OAC contraindications. When patients with documented contraindications were excluded from analysis, post-BPA phase compliance improved from 72% to 83%. Therefore, failure to identify these patients would have led to an underestimation of the OAC compliance in our system. It also created an opportunity to identify patients who might otherwise be candidates for alternative therapies such as left atrial appendage occlusion.

The CPM implementation had a significant impact on both the management of and documentation related to OAC therapy. We observed a significant increase in CHA_2_DS_2_-VASc documentation after the CPM implementation (from 5% before CPM to 40% after CPM). The BPA had a particularly pronounced effect because it not only alerted providers when an eligible patient with AF was not on OAC but also facilitated real-time documentation of contraindications. Moreover, the CPM’s use of a BPA-linked dropdown menu enabled us to capture not only clinical contraindications (eg, bleeding risk, fall risk) but also patient preferences (eg, refusal), which are central to real-world and shared decision-making frameworks.

After its introduction, documentation of contraindications increased from 0% in the pre-BPA phases (phases 1 and 2) to 11% in the post-BPA phase (phase 3). Notably, the improvement in OAC prescription rates occurred independently of this documentation, given that the unadjusted compliance rate increased significantly from 66.4% to 72.1% between the post-CPM and post-BPA phases (phases 2 and 3, respectively). These findings suggest that a well-designed, alert-based clinical decision support tool can meaningfully influence provider behavior. This observation aligns with findings from Piazza et al^13^ who demonstrated that the use of a BPA increases the likelihood of OAC prescription among patients with AF in the outpatient setting.

Our study also revealed a modest but statistically significant increase in cardioversion procedures performed in the ED after CPM implementation. Although we cannot infer causality owing to the observational nature of the study, this increase is plausibly linked to the CPM’s emphasis on timely rhythm control and structured decision making. Promoting cardioversion in appropriate patients was a core focus of the CPM, supported by establishing a dedicated AF response team that facilitated rapid evaluation, eligibility assessment, and coordination of care. The observed increase likely reflects greater alignment between ED and EP providers, improved access to procedural support, and a standardized approach that encouraged earlier consideration of rhythm control strategies.

Several studies have evaluated the impact of CPM’s on LOS and admission rates among patients with AF presenting to the ED, yielding mixed results.^11^^,^^12^^,^^20^, ^21^, ^22^ Most of these studies found that these algorithms reduced admission rates for patients with AF.^11^^,^^12^^,^^20^^,^^21^ In our study, the admission rate for patients with AF decreased from 44% to 38% after the CPM implementation. It is plausible that this drop in admission rates was influenced by the CPM implementation. Factors that may have influenced this decrease in admissions include greater access to definitive therapy in the ED, such as cardioversion, and the knowledge that patient could be seen in a dedicated AF clinic within 7 days of discharge.

Regarding ED LOS, 2 of the abovementioned studies reported an increase in LOS, whereas 1 found no difference.^11^^,^^20^^,^^21^ This is in keeping with our findings that demonstrated a modest but statistically significant increase in LOS for ED patients after the CPM implementation. We cannot exclude the possibility that the introduction of a new workflow for the treatment of AF in the ED might have led to increased LOS. For example, it is conceivable that the emphasis on cardioversions might have led to additional diagnostic testing (eg, transesophageal echocardiogram) and procedure time (direct current cardioversion), which could theoretically affect LOS. In addition, the coronavirus disease 2019 pandemic significantly disrupted hospital operations across Hawaii from early 2020 to 2022, resulting in substantial strain on our health care system. These disruptions included increased patient volumes, staffing shortages, higher-acuity patients requiring more complex care, and inpatient bed shortages, all of which may have affected LOS. There was also a nonsignificant trend toward increased LOS for patients who were admitted to the hospital.

Our study distinguishes itself from previous similar investigations in several key aspects, particularly in terms of its comprehensive scope. Although previous studies have generally concentrated on isolated metrics such as admission rates, LOS, or OAC rates, our analysis evaluated multiple outcomes simultaneously, all relevant to AF management. This multidimensional approach offers a broader perspective on AF care within the ED setting and highlights the impact of a clinical pathway model (CPM) across several domains of care.

The breadth of our analysis was facilitated by the development and implementation of a sophisticated data analytics portal. This innovative tool enabled near-real-time examination of multiple metrics, allowing for a more dynamic and responsive evaluation of the ED CPM for AF. The ability to analyze data in near real time represents a significant advancement in the field, offering potential for more agile and adaptive care strategies.

Our assessment spanned a 21-month baseline period followed by a 39-month postintervention phase—longer than in most published investigations. This extended timeframe surpasses the duration of many comparable studies in the field, allowing for a more comprehensive evaluation of long-term trends and the ability to measure the sustained impact of the CPM. To the best of our knowledge, this study represents one of the most comprehensive AF ED CPMs published to date. Our findings suggest that a properly designed and implanted CPM can have a meaningful and sustained impact on physician behavior.

There are several limitations to our study. First, because this is an observational study, there are inherent limitations with respect to inferring causality. As a result, we can only conclude that our findings are hypothesis generating because they were not the result of a randomized design. Furthermore, we cannot exclude the potential influence of broader secular trends—including increased provider familiarity with direct oral anticoagulants, evolving guideline recommendations, or parallel quality initiatives within our system—that may have contributed to the observed improvements.

Regarding readmission rates, although we did assess 30-day readmission rates within our health system, we were not able to capture admissions to hospitals outside of our network, which we acknowledge as a limitation. However, given that there was no significant difference in readmission rates between the pre- and post-CPM periods, we believe that this limitation is unlikely to have meaningfully influenced our primary findings.

In addition, the onset of the global pandemic was markedly disruptive to our operations, and it is difficult to ascertain what our results would have looked like in its absence. Finally, the study was conducted within a single health care system in Hawaii. The effectiveness of the CPM may vary in different geographic locations or health care systems with different patient populations, resources, or practice patterns.

In conclusion, we report the results of a comprehensive CPM for patients presenting with AF throughout the QHS in Hawaii. We were able to demonstrate that a streamlined approach to AF management can result in increased compliance with respect to OAC and a more accurate understanding of OAC compliance by documenting contraindications in a centralized data portal. We were also able to increase the rate of cardioversions taking place in the ED and reduce admission rates. Our CPM seemed to increase ED LOS but had no significant impact on inpatient LOS.

## Disclosures

The authors have no conflicts of interests to disclose.
